# Potential Risks Associated with the Proposed Widespread Use of Tamiflu

**DOI:** 10.1289/ehp.9574

**Published:** 2006-10-11

**Authors:** Andrew C. Singer, Miles A. Nunn, Ernest A. Gould, Andrew C. Johnson

**Affiliations:** 1 Centre for Ecology & Hydrology, Oxford, United Kingdom; 2 Centre for Ecology & Hydrology, Wallingford, United Kingdom

**Keywords:** antiviral, avian influenza, bird flu, catchment model, oseltamivir, pandemic, pollution, viral resistance, Tamiflu, wildfowl

## Abstract

**Background:**

The threat of pandemic influenza has focused attention and resources on virus surveillance, prevention, and containment. The World Health Organization has strongly recommended the use of the antiviral drug Tamiflu both to treat and prevent pandemic influenza infection. A major concern for the long-term efficacy of this strategy is to limit the development of Tamiflu-resistant influenza strains. However, in the event of a pandemic, hundreds of millions of courses of Tamiflu, stockpiled globally, will be rapidly deployed. Given its apparent resistance to biodegradation and hydrophilicity, oseltamivir carboxylate (OC), the active antiviral and metabolite of Tamiflu, is predicted to enter receiving riverwater from sewage treatment works in its active form.

**Objective:**

Our objective in this study was to determine the likely concentrations of OC released into U.S. and U.K. river catchments using hydrologic modeling and current assumptions about the course and management of an influenza pandemic.

**Discussion:**

We predict that high concentrations of OC (micrograms per liter) capable of inhibiting influenza virus replication would be sustained for periods of several weeks, presenting an increased risk for the generation of antiviral resistance and genetic exchange between influenza viruses in wildfowl. Owing to the apparent recalcitrance of OC in sewage treatment works, widespread use of Tamiflu during an influenza pandemic also poses a potentially significant, uncharacterized, ecotoxicologic risk in each affected nation’s waterways.

**Conclusion:**

To gauge the hazard presented by Tamiflu use during a pandemic, we recommend *a*) direct measurement of Tamiflu persistence, biodegradation, and transformation in the environment; *b*) further modeling of likely drug concentrations in the catchments of countries where humans and waterfowl come into frequent close contact, and where significant Tamiflu deployment is envisaged; and *c*) further characterization of the risks of generating Tamiflu-resistant viruses in OC-exposed wildfowl.

Since 2003, > 51 countries have confirmed the presence of type-H5 avian influenza in animals. According to the Food and Agriculture Organization (FAO) > 220 million poultry have been culled since the end of 2003 to control the spread of the virus (FAO 2006). The World Health Organization (WHO) confirmed 41 human deaths from avian influenza A (AIA) H5N1 in 2005, and 74 people have already died in 2006, more than twice the pace of the previous year (WHO 2006a, 2006b). The current state of alert provides an unprecedented opportunity for the global community to devise strategies to decrease mortality rates in the event of pandemic influenza. An integral component of these strategies is the manufacture of an effective vaccine. However, this cannot be produced until the emergence and identification of the pandemic influenza strain (McKimm-Breschkin et al. 2003). During the time it will take to prepare a vaccine, the WHO (2006) recommends Tamiflu [oseltamivir phosphate (OP); Figure 1] for the treatment and prevention of pandemic influenza. Tamiflu, produced and marketed by Hoffmann-La Roche Inc. (Nutley, NJ, USA), is a rationally designed selective inhibitor of influenza A and B neuraminidase (NA) (Kim et al. 1997; Li et al. 1998; von Itzstein et al. 1993). After absorption in the gastrointestinal tract, Tamiflu is converted to the active NA inhibitor oseltamivir carboxylate (OC) by hepatic esterases (Figure 1, Table 1). OC binds tightly to the highly conserved active site of the viral NA (Air and Laver 1989; Baker et al. 1987), thereby inhibiting the action of the enzyme that is needed for release of progeny virions from the surface of infected cells (Bardsley-Elliot and Noble 1999; Palese and Compans 1976).

Stockpiles of Tamiflu have been amassed by many nations worldwide to be used in the event of an influenza pandemic (in millions of treatment courses): Belgium, 3; France, 14; Germany, 16; Greece, 0.2; Italy, 30; Netherlands, 5; New Zealand, 0.84; Russia, 150; Spain, 10; United Kingdom, 14.6; and United States, 81 (BBC 2006). The WHO and the United States have stockpiled an additional 5–6 million courses combined for the purpose of “blanketing” regions upon confirmation of an outbreak (F. Hoffman-La Roche Ltd. 2006). The ring or blanketing prophylaxis strategy, an approach for delivering large amounts of antiviral drugs to people within a defined area surrounding a limited localized influenza outbreak, will greatly increase the overall use of Tamiflu in a region compared with earlier strategies that aimed to treat only clinically infected cases (Ferguson et al. 2006). Plans for antiviral stockpile deployment are country specific because of the variations in demographics, infrastructure, and available stockpiles. However, most plans are structured around WHO preparedness plans (WHO 2006c).

In the event of a human outbreak, the current WHO strategy (WHO 2006c) recommends that all infected individuals (> 1 year of age) receive a 5-day course of 750 mg Tamiflu (i.e., 75 mg twice per day for 5 days). This quantity will also be needed for a 10-day prophylaxis course (i.e., 75 mg once per day for 10 days), which could be extended for several weeks until there is no further risk of exposure to AIA. Given the large doses of Tamiflu, it is highly significant that approximately 70% of each oral dose is excreted renally in the active antiviral form, OC, with up to 20% appearing in the feces (50% as OP and 50% as OC; Table 1) (He et al. 1999). Hence, up to 80% of an oral dose of OP can be excreted as OC. No observed oxidative metabolites of OC have been reported in humans, indicating that OC is resistant to cytochrome P450 mixed-function oxidases and glucuronosyltransferase (He et al. 1999; Sweeny et al. 2000). OC has not been reported to undergo appreciable mineralization based on a standard Organization for Economic Co-operation and Development (OECD) biodegradability test [European Medicines Agency (EMEA) 2005]. Hence, the active antiviral, OC, has the potential to be maintained in rivers receiving treated wastewater.

The acute timescales involved in pandemic treatment and containment and the biophysicochemical characteristics of Tamiflu prompted us to ask whether the concentration of OC in river water presents an ecotoxicologic risk or a pharmacologically relevant risk to hastening the development of Tamiflu resistance in wildfowl.

## Methods and Discussion

Tamiflu was launched by Roche in North America in October 1999, but there is very little research regarding the environmental fate and toxicology of the drug. OC has both amine and carboxylate groups in the molecule imparting hydrophilicity, a low partition coefficient (log P of 1.1), and high water solubility (588 mg/mL at 25°C) (American Hospital Formulary Service 2006). These physicochemical features of OC will minimize loss by sorption to sewage sludge during wastewater treatment. In the absence of empirical evidence to the contrary, Relenza (GlaxoSmithKline, Brentford, Middlesex, UK), a structurally similar NA inhibitor, was used as a model for estimating the ecotoxicologic and environmental fate of Tamiflu. Relenza is *a*) not likely to sorb to soil or sediment if released directly into the environment; *b*) not likely to partition to fats; *c*) not readily volatile; *d*) readily soluble in water; *e*) chemically stable in water [thus, hydrolysis is unlikely to be a significant depletion mechanism (half-life > 1 year); and *f* ) is not readily mineralized (aerobic; degradation < 1% in 28 days in activated sludge) (GlaxoSmithKline 2004).

To assess the potential biodegradability of Tamiflu and OC, we used a metabolic pathway prediction system that recognizes the organic functional groups found in a compound and predicts transformations based on metabolic rules (Hou et al. 2004). Only one biotransformation for OC was generated by the program, employing an amine oxidase. The general structure of OC is not radically changed by this biotransformation, thus the OC metabolite might retain NA inhibitor properties. The only published account of the biodegradability of Tamiflu is within the documentation submitted by Roche to the EMEA (2005) in which it refers to the OECD carbon dioxide evolution test (Modified Sturm test) that investigates the mineralization of Tamiflu in an aerobic slurry enriched with sewage sludge. At the conclusion of the 28-day assay, < 20% of Tamiflu had been converted to carbon dioxide, demonstrating its recalcitrance and likely persistence in sewage treatment plants (EMEA 2005). The apparent resistance of OC to further biotransformation in the human body (He et al. 1999) and the limited biodegradation reported in carbon dioxide evolution tests (EMEA 2005), plus its structural similarity to the environmentally persistent NA inhibitor Relenza, strongly suggests that both Tamiflu and OC will persist in wastewater and river water.

We examined catchments in the United Kingdom and the United States to generate examples of possible OC water concentrations. Catchment boundaries and the human population within the United Kingdom for 1991 were provided by the computerized digital terrain network (Bracken and Martin 1989). However, from 1991 to 2004, the population of England and Wales has grown by 6.8%; therefore, we estimated the 2004 population by scaling up. We predicted the natural flow from the catchment surface area, discharge point, and geographic location, as described by Young et al. (2003). We calculated the annual mean flow and available dilution per capita from the mean annual rainfall for 1961–1991 and from assumptions on runoff and evaporation for that location (Table 2) (Holmes et al. 2002).

**Figure 2:**
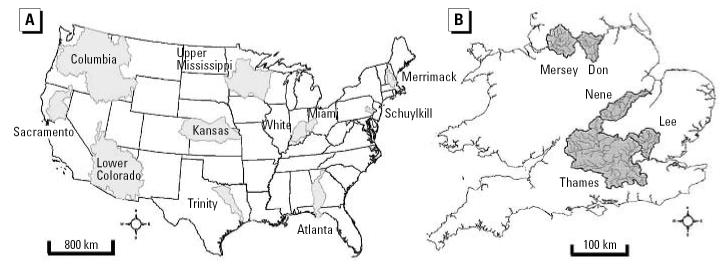
Illustration of the distribution of catchments we investigated within (*A*) the United States [adapted from Anderson et al. (2004)] and (*B*) the United Kingdom.

Eleven substantial catchments have been examined in the United States as part of a modeling exercise for pharmaceuticals (Anderson et al. 2004). These catchments were selected to represent the variety present within the United States (Figure 2A): They represent 19% of the land area, contain 14% of the human population, and receive waste from 1,112 sewage treatment plants (Anderson et al. 2004). In general, these selected American catchments are larger than those in the United Kingdom, with lower population densities, and thus have more available dilution per head of population. The Lower Colorado River is an exception, being in an arid area with very low flow. Five catchments within the United Kingdom have been selected for the modeling exercise, particularly focusing on low naturalized flow (Figure 2B).

The concentration of OC in river water on a given day is provided by the following equation:


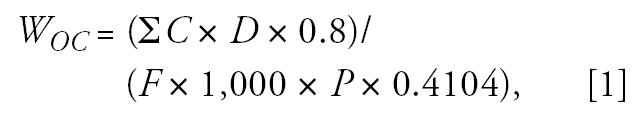


where *W_OC_* is the OC concentration (nanomolar) in river water; ∑*C* is the sum of active courses consumed by clinically infected people in a U.K. or U.S. catchment within the previous 5 days (reflecting the recommended treatment course of Tamiflu); *D* is the daily dose of Tamiflu (150,000 μg; 0.8 is the predicted maximum fraction of an oral dose of Tamiflu that is converted to OC and released into the waste-water); *F* is the available dilution per person (cubic meters per day per person); and *P* is the population in the catchment. For the United Kingdom and the United States, we used the clinical case incidence per day suggested by Ferguson et al. (2006), in which they modeled a virus with a high or moderate basic reproductive number (R_0_) of either 2 or 1.7. R_0_ is the average number of secondary infections produced by an infected individual in a fully susceptible population (Anderson and May 1991). We divided the number of clinically infected people per day by the respective total population to yield the fraction of the population infected on a daily basis. This fraction was used to estimate daily numbers of clinically infected people for each catchment. In reality, a range of concentrations would occur across the different reaches of a catchment. However, the mean concentrations from which naturalized flow is based indicate concentrations that could be expected and also allow comparisons to be made across different catchments. Multiplying the naturalized flow by 1,000 converts the units to liters of river water; to convert units from micrograms of OP per liter to nanomolar OC requires division by 0.410.

In brief, the model assumes that *a*) all clinical cases were treated at the first sign of infection with a full course of Tamiflu lasting 5 days, with 100% compliance; *b*) Tamiflu was used only by clinically infected people, as determined by Ferguson et al. (2006); *c*) 80% of the ingested Tamiflu was released as OC; and *d*) all of the OC entering the catchment was flushed out in 1 day (an underestimate for most of these catchments). The decision to use only clinically infected people in the model ensures a conservative estimate of OC in river-water because the model omits prophylactic use and personal stockpiles; the latter was omitted because the extent of personal stockpiling and the quality of drug being stockpiled is unknown. Depending on how the pandemic might develop, prophylaxis might increase projections by > 100%.

**Figure 3:**
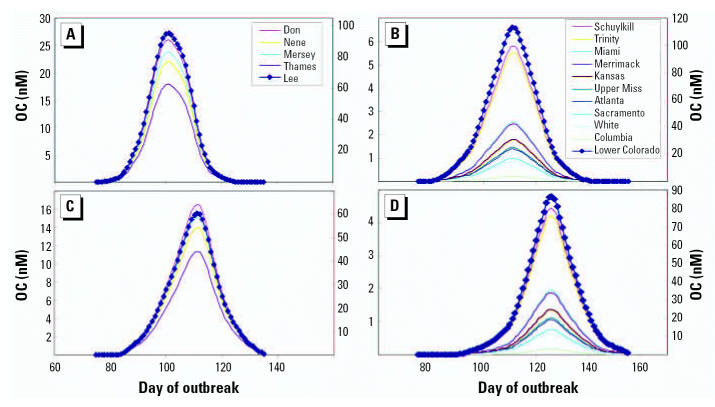
Predicted concentration of OC in UK (*A, C*) and U.S. (*B, D*) rivers generated from a population with a cumulative total clinical infection rate of 35% (R_0_ = 2.0; *A, B*) or 25% (R_0_ = 1.7; *C, D*), assuming a generation time of 2.6 days and that 50% of infected people are sufficiently ill to be classified as clinical cases. Refer to the right-hand *y*-axes for values for Lee (*A, C*) and Lower Colorado (*B, D*). The day of outbreak refers to the days after a global influenza outbreak as a function of the expected importation of infection from overseas [as per Figure 1A and B in Ferguson et al. (2006)].

Figure 3 shows broad estimates (based on the average flow prediction) of the concentration of OC in river water over the time course of an epidemic for viruses with moderate or high transmission (R_0_, 1.7 or 2.0). Table 2 provides estimates for the number of consecutive days that OC is projected to exceed 1 nM and 50 nM. As highlighted in Figure 3A, OC concentrations in the Lee catchment (R_0_ = 2.0) exceed 95 nM for nearly 1 week. Similarly, we predict that the Lower Colorado catchment (Figure 3A) would have river water concentrations > 90 nM for 10 days, reaching a maximum concentration of 112 nM (R_0_ = 2.0; Figure 3B). The Colorado catchment is predicted to contain > 50 nM OC for 62 consecutive days, notably in the lower transmission level conditions (R_0_ = 1.7; Figure 3D). All five catchments investigated in the United Kingdom are predicted to have > 34 consecutive days with OC > 1 nM (R_0_ ≥ 1.7). Owing to lower population density in many of the U.S. catchments, the peak concentrations achieved during a pandemic are approximately 10 times less than U.K. river water OC levels. However, of the U.S. catchments modeled, 9 of 11 attained river water OC levels > 1 nM when R_0_ = 2.0 (Figure 3B) and 8 of 11 catchments when R_0_ = 1.7 (Figure 3D).

A river water OC concentration of 1 nM is 1.6–625 times higher than three commonly prescribed drugs in U.K. river water: *a*) the oral contraceptive ethinylestradiol at 1.6 pM (Johnson et al. 2006); *b*) the pain killer diclofenac at 139 pM (Johnson et al. 2006); and *c*) the beta blocker propranolol at 594 pM (Ashton et al. 2004). Although not discussed in any detail here, the range of OC concentrations predicted in river water may have uncharacterized ecotoxicologic consequences. Based on the structure–activity relationship determined using toxTree (version 1.00; Ideaconsult Ltd., Sofia, Bulgaria), both Tamiflu and OC are predicted to be “class 3” toxicologic hazards. ToxTree employs the Cramer decision tree approach, relying primarily on chemical structures to estimate toxic hazard to establish priorities for more in-depth toxicologic testing (Cramer et al. 1978). In an acute toxicity study in *Daphnia magna*, OP was classified as harmful according to the European Union Directive 67/548/EEC (EMEA 2005). The EMEA documentation on the ecotoxicity of Tamiflu states,

Considering ecotoxicological properties, use pattern, dosage and maximal estimated amounts of oseltamivir to be placed on the market, no exposure levels of concern to the environment are to be expected. (EMEA 2005)

However, it is unclear whether this ecotoxicologic risk assessment for OP was intended to apply to pandemic avian influenza conditions. In summary, we raise a number of, as yet, unconsidered risks to the environment from Tamiflu use during a pandemic, most notably, the ecotoxicologic hazard associated with the release of a uniquely structured, biochemically resistant antiviral drug. The recalcitrance of OC to metabolism in humans, sewage treatment works, and river water will enable IC_50_ (concentration that causes 50% inhibition)-relevant concentrations to be reached in catchments, potentially influencing the generation of Tamiflu-resistant AIA in wildfowl.

The avian species most commonly infected with AI are wildfowl of the order Anseriformes (e.g., ducks, geese, swans) (Alexander 2000). The AIA virus is believed to be transmitted between waterfowl by the fecal–oral route as they imbibe contaminated water, although in a recent report with one AIA strain, Normile (2006) demonstrated that the virus was shed far more heavily in the duck pharynx than through the feces. In birds, the virus replicates in the lower intestinal tract (small and large intestine) and in the lungs. In the gut it buds from the surface of mucosal cells into the lumen (Webster et al. 1978). In the event of widescale Tamiflu use, waterfowl would ingest large quantities of active OC together with virus in their daily water intake, ranging from 64 mL/kg/day in glaucous-winged gulls (Walter and Hughes 1978) to 200–300 mL/kg/day in duck species (Hughes 2003). Because of the poor bioavailability of OC relative to its prodrug, Tamiflu (Kim et al. 1997; Li et al. 1998), a high percentage of OC ingested by avian species will remain in the intestinal tract, the primary site of viral replication in Anseriformes. Waterfowl have been shown to reabsorb urine into the rectum, ileum, and/or ceca, amounting to as much as 40% of a mallard duck’s daily water influx, thereby concentrating nontransported ions and molecules in the lumina (Hughes et al. 1999). Hence, the concentration of OC in the gut of waterfowl might be higher than that found in the riverwater. The published IC_50_ of the NA enzyme for OC varies widely depending on the assay method and AIA isolate, ranging from 0.01 to 114.0 nM OC (Ferraris et al. 2005; Hurt et al. 2004; Mendel et al. 1998; Roche 2004). Le et al. (2005) reported the IC_50_ for drug-resistant H5N1 virus (A/Hanoi/30408/2005) to be 90 nM.

The projected concentrations of OC in river water (Table 2) could therefore drive the selection of resistant strains of AIA in the gut of waterfowl. Notably, it is relatively easy to generate Tamiflu-resistant mutants because single amino acid substitutions in NA have conferred high-level OC resistance (Colacino et al. 1997; de Jong et al. 2005; Le et al. 2005; McKimm-Breschkin et al. 2003; Mendel and Sidwell 1998). Furthermore, the gradual rise of OC to IC_50_ and IC_90_-relevant concentrations during the course of an AIA epidemic, as predicted here, would be more effective in generating Tamiflu-resistant mutants than would a sudden spike. A similar gradual increase is used to select resistant mutants in laboratory experiments.

## Conclusions

We recognize that a highly pathogenic human-to-human transmissible AIA strain is unlikely to be frequently transmitted between humans and waterfowl (Russell and Webster 2005). However, the possibility of promoting new OC resistant AIA strains in wildfowl introduces another important factor to be considered in our international AIA strategy. This is particularly important given the mobility of migrating wildfowl. Occasional co-infection in wildfowl could generate new strains incorporating Tamiflu-resistant NA genes. This scenario is most likely to occur in southeast Asian countries where humans and waterfowl frequently come into close direct or indirect contact, and where significant Tamiflu deployment is envisaged. We therefore recommend similar, but more detailed, modeling exercises in such countries to assess the potential environmental and virologic risks that we have highlighted. It will also be important to identify the locations that provide the greatest intersection between human waste-water effluent and waterfowl migration within relevant countries. More detailed studies need to be conducted to identify the extent of biotransformation that OC might undergo in different environments, in addition to assessing the susceptibility of OC to photodegradation. Attention should be given to developing methods to minimize the release of OC into the waste stream, such as biological and chemical pretreatment in toilets, which could eliminate much of the “downstream” risk. Regulatory guidance might be needed to ensure proper disposal of the Tamiflu stockpiles once they expire.

## Figures and Tables

**Figure 1:**
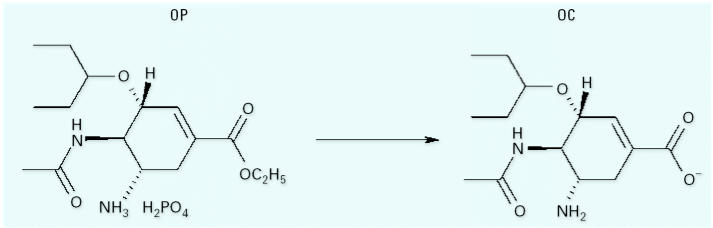
Structure of the prodrug Tamiflu (OP; CAS Registry no. 204255-11-8; molecular weight, 410.4) and the active form OC (CAS Registry no. 187227-45-8; molecular weight, 284.35).

**Table 1:** Summary of mean pharmacokinetic values for absorption, distribution, metabolism, and elimination of Tamiflu (OP) and the active antiviral OC (Li et al. 1998) as a proportion of a single 75-mg oral dose (He et al. 1999).

			Excreted				
	Blood		Urine		Feces		
	Total-OC	Protein bound	OP	OC	OP	OC	Total fraction excreted
75-mg dose	0.8	0.03	0.05	0.7	0.1	0.1	0.95

**Table 2:** Populations, naturalized flows, and projected number of days with significant concentrations of antiviral in catchments in the United Kingdom and the United States.

							Days with > 1 nM OC		Days with > 50 nM OC	
Catchment	Location	Population (2004)	Flow (m^3^/day)	Area (km^2^)	Population density/km^2^	Dilution (m^3^/head/day)	R_0_ = 2.0	R_0_ = 1.7	R_0_ = 2.0	R_0_ = 1.7
Lee	Northeast London, UK	1,777,126	580,003	1,412	1,258	0.3	43	50	16	9
Don	South Yorkshire, UK	1,309,305	1,436,832	1,305	1,003	1.1	36	44	0	0
Mersey	Lancashire, UK	2,860,635	3,405,024	2,043	1,400	1.2	36	42	0	0
Nene	Northamptonshire, UK	631,680	813,024	1,799	351	1.3	36	43	0	0
Thames	Southern England, UK	4,430,918	7,001,856	9,959	445	1.6	34	41	0	0
Lower Colorado	Southwest USA	5,861,200	1,223,424	350,060	17	0.2	58	62	23	16
Schuylkill	Northeast USA	1,950,400	7,661,081	5,000	390	3.9	35	31	0	0
Trinity	Southern USA	5,104,300	20,864,273	46,540	110	4.1	35	30	0	0
Miami (Ohio)	Central USA	1,809,700	16,154,738	13,900	130	8.9	25	19	0	0
Merrimack	Northeast USA	2,090,300	19,315,714	13,030	160	9.2	25	12	0	0
Kansas	Central USA	1,333,700	16,834,314	155,660	9	12.6	21	8	0	0
Upper Mississippi	Central USA	5,291,500	82,537,673	174,735	30	15.6	16	6	0	0
Atlanta headwaters	Southern USA	3,894,400	63,791,774	52,860	74	16.4	15	0	0	0
Sacramento	Central West USA	2,589,100	59,365,426	72,260	36	22.9	5	0	0	0
White	Central USA	2,465,600	65,281,975	31,600	78	26.5	0	0	0	0
Columbia	Northwest USA	6,306,400	639,894,795	570,135	11	101.5	0	0	0	0

R_0_, basic reproductive number.
